# EXAMINING THE COMPLETENESS OF MEDICARE ADVANTAGE ENCOUNTER DATA FOR MEASURING POST-ACUTE CARE UTILIZATION

**DOI:** 10.1093/geroni/igae098.4311

**Published:** 2024-12-31

**Authors:** Hyunkyung Yun, Cyrus Kosar

**Affiliations:** Brown University, Providence, Rhode Island, United States; Brown University, Providence, Rhode Island, United States

## Abstract

Given the rapid growth of Medicare Advantage (MA) program, the degree to which the newly-released MA encounter data reliably capture care utilization has serious implications for the ability to evaluate MA-related policies accurately and conduct rigorous academic research. However, little is known about the quality of MA encounter data outside of the acute care setting. We assessed how well the encounter data captured post-acute skilled nursing facility (SNF) and home health (HH) utilization overall and across MA contracts. We identified 2016-2019 Minimum Data Set (MDS) and the Outcome and Assessment Information Set (OASIS) admission assessments preceded by inpatient claims and calculated the proportion of assessments matched to a SNF or HH encounter record within 7 days of the assessment’s admission date. In 2019, there were 871,101 MDS assessments and 812,002 OASIS assessments following an MA inpatient stay. About 82% of MDS assessments could be matched to a SNF encounter records (up from 75% in 2016) while 63% of OASIS assessments matched to a HH encounter record (52% in 2016). There was substantial contract-level variation in match rates and a lack of correlation across services. Only 21% of contracts that had high SNF encounter completeness (match rates of 90% and above) also had high HH completeness, and 68% of contracts with high HH completeness also had high SNF completeness. Efforts to improve the quality of MA encounter data are needed as CMS prioritizes this data as a source for program oversight and payment.

